# Rotation Culture of Macroalgae Based on Photosynthetic Physiological Characteristics of Algae

**DOI:** 10.3390/biology13060459

**Published:** 2024-06-20

**Authors:** Xiaopeng Cheng, Xu Zhao, Jun Lin, Shouyu Zhang, Zhenhua Wang, Hong Huang, Kai Wang, Jianqu Chen

**Affiliations:** 1 College of Marine Ecology and Environment, Shanghai Ocean University, Shanghai 201306, China; xpcheng@shou.edu.cn (X.C.); xzhao@shou.edu.cn (X.Z.);; 2 East China Sea Fisheries Research Institute, Chinese Academy of Fishery Sciences, Shanghai 200090, China

**Keywords:** macroalgae, rotation culture, photosynthetic characteristics, ecological effect, physiological suitability

## Abstract

**Simple Summary:**

Seaweed aquaculture plays an important role in global food supply, but single-species intensive seaweed aquaculture has problems related to simple industrial structure and risk susceptibility. Therefore, there is an urgent need to establish a series of large-scale seaweed rotational culture models to address the current industrial challenges in algae culture. The culture of macroalgae is mainly affected by environmental factors such as water temperature and light. In this study, we constructed a rotational culture model by carrying out experiments with three species of macroalgae, observing in situ their photosynthetic activity responses to different temperature and light conditions. The results of this study can provide a theoretical basis for the establishment of large-scale rotational aquaculture, which can effectively improve the ecological and economic value of macroalgae aquaculture.

**Abstract:**

Seaweed farming has made outstanding contributions to food supply and the restoration of the ecological environment despite the limitations in production and ecological effects due to the current intensive farming of single algae species. These limitations can be overcome by selecting suitable algal species based on their physiological characteristics and by constructing a large-scale seaweed rotation model. This study carried out a trial culture in aquaculture sea areas, and performed in situ monitoring of the environmental conditions and physiological characteristics of *Saccharina japonica*, *Hizikia fusiformis*, and *Gracilariopsis lemaneiformis*. Additionally, a comparative analysis of the three macroalgae at different times was conducted to determine their response characteristics to environmental factors. The results showed that: (1) The three macroalgae had varying light tolerance. The effective quantum yield of *Hizikia fusiformis* and *Gracilariopsis lemaneiformis* remained unchanged during the changes in light environment, while that of *Saccharina japonica* first decreased and then recovered. (2) The relative electron transport rates of the three macroalgae were significantly different under different temperature conditions. *Hizikia fusiformis* and *Saccharina japonica* exhibited the highest relative electron transport rates (70.45 and 106.75, respectively) in May (20.3 °C). Notably, *Gracilariopsis lemaneiformis* demonstrated good growth and exhibited the highest relative electron transport rate (93.07) in September (27.5 °C). These findings collectively support the feasibility of establishing a macroalgae rotation model. Based on the combined environmental conditions of the seas in Shandong, Zhejiang, and Fujian, a macroalgae rotation model was proposed. The application of this model in the construction of artificial seaweed farms in marine ranches can provide a stable output of large-scale seaweed production and ecological benefits.

## 1. Introduction

Algae farming contributes significantly to solving global food demand and ecological challenges. According to statistical reports by FAO [1], the global seaweed production in 2018 was 3180 Mt (wet weight), accounting for 51.3% of the global marine aquacultural production and contributing to about USD13.3 billion in income for farmers [2,3]. In addition to meeting the market food demand, large seaweeds also provide high-quality feeding grounds for the cultivation of invertebrates, such as abalone, and provide abundant raw materials for the extraction of various products, such as fucoidan and mannitol. Macroalgae also act as biofilters, playing an essential role in addressing environmental and ecological problems [4]. At present, many studies have shown that large-scale seaweed culture plays an important ecological role in regulating water eutrophication, alleviating ocean acidification, increasing marine carbon sequestration, and providing a habitat for fishery resources [5,6,7,8,9,10].

China’s large-scale algae culture is dominated by cold-water algae. The total area of algae culture in China in 2018 was 1.44 × 10^5^ hm^2^, whose entire output reached 2.34 million tons (wet weight) (China Fishery Statistical Yearbook 2019). For example, the raft *Saccharina japonica* culture structure has solved its demand in light conditions. The technical breakthrough to produce summer seedlings in cooled greenhouses has successfully promoted *Saccharina japonica* culture in the North (N 36°) [11]. Similarly, the high-temperature-resistant kelp varieties, cultivated through gametophyte hybridization technology, have enabled the rapid kelp culture in the south (N 23°) [12]. Currently, the area dedicated to *Saccharina japonica* culture accounts for about 31% of the area available for algae culture. Of note, the culture cycle of large algae is limited by the environmental water temperature. For example, the culture cycle of *Saccharina japonica* is within the time range when the water temperature is below 20 °C [13,14,15]. The intensive cultivation of a single algae species is marked by a simple industrial structure and limited resilience to risks [16]. In a seaweed cultivation area dominated by cold-water algae species, a common issue is the occurrence of cultivation vacancies during high-temperature conditions in summer, leading to time and space limitations in the yield and ecological utility of large algae. Therefore, it is urgent for researchers to identify suitable algae species, increase the diversity of cultured algae species, and fill the gap in seaweed culture during summer. Establishing large-scale seaweed rotation culture models can help coordinate the supply and demand of individual physiological and environmental rhythms of cultured algae species, improving the viability and stability of the algae culture industry.

The process of photosynthesis is an important target of abiotic stress. Photosynthetic performance is thus widely used as an index to evaluate the physiological adaptability of algae to environmental conditions [17]. The chlorophyll fluorescence technology is a rapid, non-invasive, sensitive, and convenient method for monitoring the photosynthetic performance of autotrophs. It allows us to assess such critical photosynthetic parameters as photosystem II (PSII) light energy absorption, electron transport from PS II to PSI, non-photochemical fluorescence quenching, and others [18,19]. To date, the technology has been widely used in laboratory physiological research. Many researchers have studied the physiological response of large algae to environmental changes, such as global warming and ocean acidification. These studies provide a theoretical basis for understanding the tolerance and ecological suitability of macroalgae to abiotic factors [20,21,22,23,24,25,26,27,28,29,30,31,32,33,34].

The environmental conditions of large-scale algae cultivation in the field are complex and diverse. Previous physiological experiments primarily focused on observations under controlled indoor conditions, which limits their ability to explain the physiological characteristics of macroalgae in the complex and diverse marine environment. This study used *Saccharina japonica*, *Hizikia fusiformis*, and *Gracilariopsis lemaneiformis* as examples to screen suitable algae species under different temperature conditions and establish an ecological rotation model of macroalgae. The field trial breeding experiment was conducted in Xihu harbor, a typical macroalgae breeding area in the inner bay of Zhejiang Province. The photosynthetic characteristics of the three macroalgae species were observed in situ using a modulated fluorescence instrument, Diving-Pam (Walz GmbH, Effeltrich, Germany), in January (winter), May (spring), and September (summer). The physical and chemical indexes of the environment in the aquaculture area were monitored synchronously. An ecological rotation culture model of large algae was established based on the characteristics of water temperature changes in different culture areas to identify the photosynthetic physiological characteristics of the three kinds of cultured macroalgae in different seasons. The model was also used to explore the possibility of applying a large-scale seaweed rotation culture model in algae culture and ecological restoration engineering.

## 2. Materials and Methods

### 2.1. Monitoring Site

**Figure 1:**
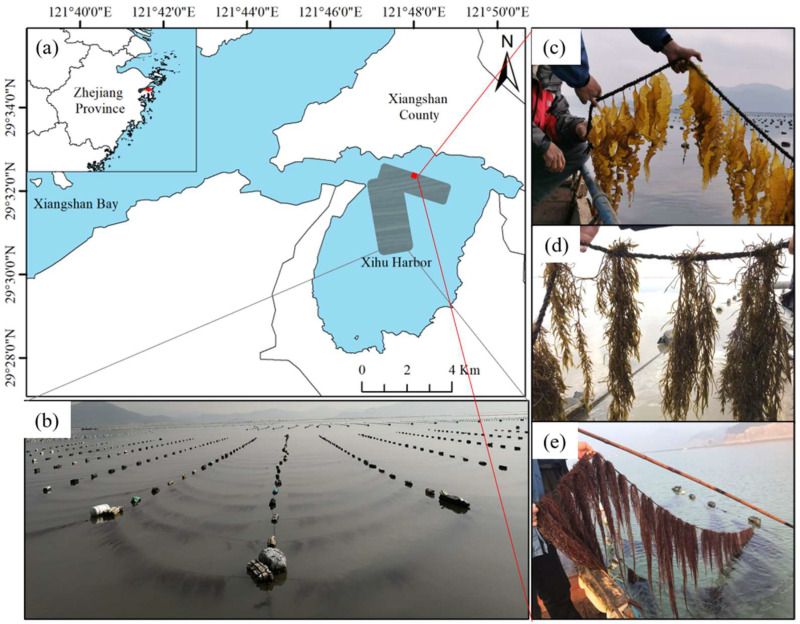
Example of macroalgae rotation in Xihu harbor. (**a**) Locations of example sites; (**b**) kelp of culture; (**c**) *Saccharina japonica*; (**d**) *Hizikia fusiformis*; and (**e**) *Gracilariopsis lemaneiformis*.

Xihu harbor (29°32′20.06″ N, 121°47′28.04″ E) is located in the southeast of Xiangshan Bay, Ningbo City, Zhejiang Province (Figure 1a). The site is a typical subtropical semi-enclosed bay. The average tidal range of Xihu harbor is about 3 m. The large-scale seaweed culture in Xihu harbor mainly includes *Saccharina japonica* and *Pyropia yezoensis*, with the former covering approximately 200 hectares. The seedlings are clamped in December of each year, and the harvest begins in April of the following year. The surface velocity of the *Saccharina japonica* culture area during spring tide is 10.63–82.48 cm/s. Notably, Xihu harbor is extensively eutrophicated due to the low water exchange rate, industrial and agricultural sewage discharge, and excessive mariculture [28].

### 2.2. Seaweed Farms

The *Hizikia fusiformis* culture experiment began in 31 October 2019. The seedlings, sourced from the Dongtou *Hizikia fusiformis* culture area in Zhejiang Province measured 24.6 ± 14.93 cm. The *Gracilariopsis lemaneiformis* cultivation experiment began in 25 November 2019. The seedlings were sourced from Qingdao, Shandong Province (provided by Mr. Tao Liu). The *Saccharina japonica* culture experiment began in 20 December 2019, with seedlings sourced from Xiapu, Zhejiang Province, measuring 37.6 ± 0.87 cm long.

The structure of the breeding raft primarily comprises a long-line and cables. The long-lines are 50 m in length and are spaced 3.5 m apart (Figure 1b). The seedling ropes, each measuring 3.8 m in length, are positioned 1.5 m apart from each other. *Hizikia fusiformis* (Figure 1d) and *Gracilariopsis lemaneiformis* (Figure 1e) have the same cultivation density, with algal bodies spaced 15–20 cm apart. For *Saccharina japonica*, the algal bodies are distanced 10–15 cm from each other (Figure 1c).

The physiological activities of the macroalgae were monitored at the same time (11:00–15:00) on January 9 (winter), May 6 (spring), and September 8 (summer) 2020, respectively. Temperature and light were also monitored synchronously. In situ monitoring of the photosynthetic activity of the macroalgae was carried out from 6 May 2020 to 7 May 2020 to understand the diurnal variation of the photosynthetic activity of the macroalgae.

### 2.3. Monitoring of Environmental Factors in Aquaculture Water

This study used a calibrated temperature, salinity, and depth multi-probe observation system (SBE 19plus V2, Sea-Bird Electronics, Bellevue, WA, USA) to monitor the surface temperature and photosynthetically active radiation in the aquaculture area of Xihu harbor. The target was to acquire the data at 0.5 m underwater after calibrating the pressure sensor. The surface and bottom water samples were collected to determine the environmental factors based on the “Ocean Monitoring Specification” (GB17378.3-2007) [35]. These samples included dissolved inorganic nitrogen nitrate (NO3-N), nitrite (NO2-N), ammonium salt (NH4-N), and active phosphate (PO4-P). The total nitrogen and total phosphorus were also measured following the “Ocean Monitoring Specification”.

### 2.4. Fluorescence Parameter Determination

The chlorophyll fluorescence parameters of the algae were monitored in situ using an underwater fluorometer, Diving-PAM, and a data acquisition system, WinControl-3 (Walz GmbH, Effeltrich, Germany). Samples (3–5) were randomly selected from the culture water layer, and the surface impurities on the algae were cleaned. An optical fiber probe was fixed with a magnetic blade clip, and the ambient light was adjusted by connecting it to a computer to maintain the light in the range of 200–500 μmol m^−2^·s^−1^. The modulation measurement light was first turned on, and the initial fluorescence value (*F*_0_′) was measured. Then, the saturation pulse was then turned on [4000 μmol m^−2^·s^−1^], a time when the fluorescence variable (*F_v_*′) was the maximum fluorescence value (*F_m_*′).

(1) $$F_{v}^{\prime }=F_{m}^{\prime }-F_{0}^{\prime }$$

The actual photochemical efficiency (*F_v_*′/*F_m_*′) could be calculated as follows:

(2) $$F_{v}^{\prime }/F_{m}^{\prime }=(F_{m}^{\prime }-F_{0}^{\prime })/F_{m}^{\prime }$$

The relative electron transport rate (*rETR*) reflects the efficiency of light energy utilization in chloroplasts. The photochemical gradient of the rapid light curve (RLC) of the three algae were 0, 99, 255, 476, 761, 1079, 1453, 2071, and 2762 μmol m^−2^·s^−1^. The irradiation time under each photochemical gradient was 10 s, while the interval between two photochemical lights was 20 s. The fluorescence value (*F_t_*) before opening the saturated pulse was recorded after each photochemical irradiation. The measured fluorescence value after opening the saturated pulse was *F_m_*′, from which the effective quantum yield of photosystem II (PSII) was calculated (effective quantum yield, *Y* (II)):

(3) $$Y\left(II\right)=(F_{m}\prime -F_{t}\prime )/F_{m}\prime$$

(4) $$rETR=Y\left(II\right)\times PAR\times 0.84\times 0.5$$

In the formula, *Y* (II) is the effective quantum yield, *PAR* is photosynthetically active radiation, 0.5 is the proportion of light absorbed with PSII, and 0.84 is the absorptivity of the sample [36].

The rapid light curve was fitted using the double exponential attenuation function and the Marquardt–Levenberg regression algorithm [18]:

(5) $$rETR={rETR}_{m}\times (1-e^{-\alpha \times PAR/{rETR}_{m}})\times e^{-\beta \times PAR/{rETR}_{m}},$$

(6) $$E_{k}=r{ETR}_{m}/\alpha$$

(7) $$E_{m}={rETR}_{m}/\alpha \times ln[(\alpha +\beta )/\beta ]$$

where *α* is the initial slope of the fast curve, *β* is the photosynthetic inhibition parameter, *rETR_m_* is the maximum relative electron transport rate, *E_k_* is the half-saturated light intensity, and *E_m_* is the saturated light intensity [18].

### 2.5. Construct Rotational Models

Based on this study, and considering the species of algae cultured in the major seaweed farming regions in China, we established a model for large-scale seaweed rotation culture. This model takes into account the annual water temperature changes at different latitudes (with observation data provided by the China Ocean Observation Station) and research findings on *Saccharina japonica*, *Undaria pinnatifida*, *Hizikia fusiformis*, *Gracilariopsis lemaneiformis,* and *Eucheuma denticulatum*. It is primarily focused on the provinces of Shandong, Zhejiang, and Fujian.

### 2.6. Statistical Analysis

All the data obtained from the experimental monitoring were sorted using the Excel software 2016, and the results were expressed as means ± standard deviation (SD). The rapid light curve data were fitted and plotted. The data distribution was tested for normality, and one-way ANOVA was used for data conforming to a normal distribution; otherwise, the Kruskal–Wallis significance test was used. The above analysis was conducted using original software.

## 3. Results

### 3.1. Changes in Environmental Factors in Aquaculture Water

**Figure 2:**
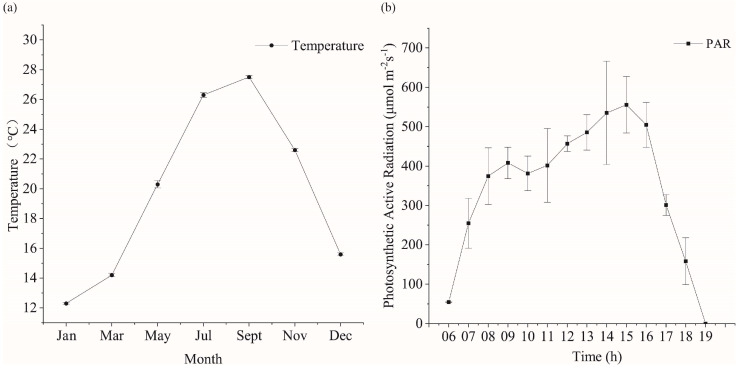
Trends of temperature and light in the trial culture. (**a**) Temperature variation of kelp culture area in Xihu harbor in different months. (**b**) Daily variation of photosynthetically active radiation in the kelp culture area of Xihu harbor.

The shift in temperature within the breeding area is predominantly noticeable across varying months, while alterations in light become apparent at different times throughout the day. The annual temperature change of the seaweed aquaculture waters of Xihu harbor is shown in Figure 2a. The minimum temperature was recorded in January (12.3 ± 0.15 °C), while the highest temperature was recorded in September (27.5 ± 0.1 °C). The daily trend of photosynthetically active radiation (*PAR*) in the seaweed farming area of Xihu harbor is shown in Figure 2b. The local sunrise time on 6 May 2020 was 5:06 a.m., the midday time was 11:50 a.m., and the sunset time was 18:34. The PAR first increased and then decreased. The maximum *PAR* was detected at around 15:00. Notably, the *PAR* had some errors because of cloud cover and other reasons.

### 3.2. Comparison of Photosynthetic Fluorescence Parameters of the Three Macroalgae

**Figure 3:**
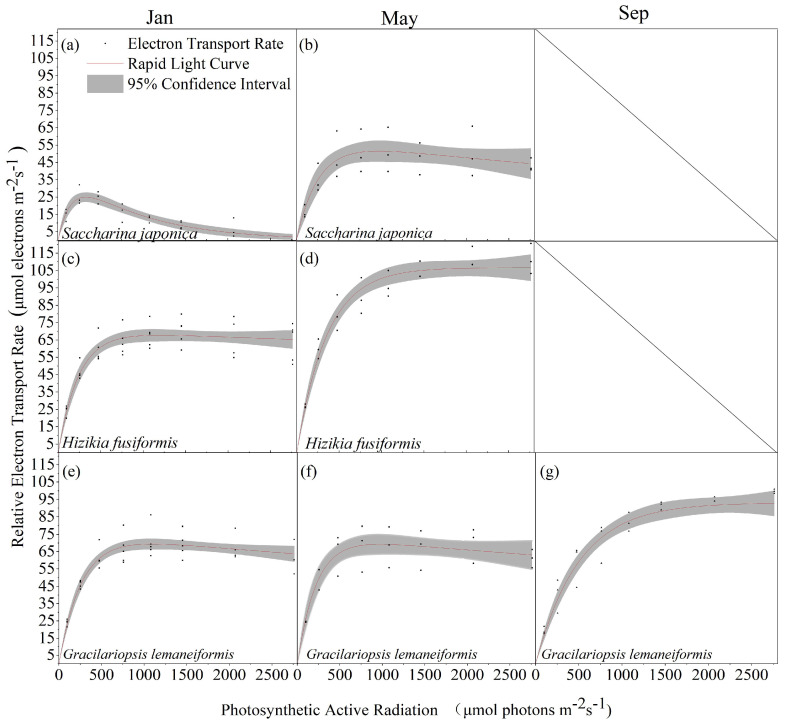
Rapid light curves of three algal species in different seasons. (**a**) *Saccharina japonica*, January; (**b**) *Saccharina japonica*, May; (**c**) *Hizikia fusiformis*, January; (**d**) *Hizikia fusiformis*, May; (**e**) *Gracilariopsis lemaneiformis*, January; (**f**) *Gracilariopsis lemaneiformis*, May; and (**g**) *Gracilariopsis lemaneiformis*, September. (*n* ≥ 3).

*Saccharina japonica* and *Hizikia fusiformis* tend to wither by September, resulting in a lack of rapid light curves. Comparison of the rapid light curves of *Saccharina japonica*, *Hizikia fusiformis*, and *Gracilariopsis lemaneiformis* at different time stages revealed that *Hizikia fusiformis* showed the maximum rETRm (106.75 μmol electrons m^−2^·s^−1^) in May (Figure 3d) and *Saccharina japonica* showed the lowest rETRm (43.58 μmol electrons m^−2^·s^−1^) in January (Figure 3a). The photosynthetic parameters of all the macroalgae changed significantly at different time stages, except for *Saccharina japonica*, which exhibited significant photo-inhibition in January (Figure 3a). The other two algae did not exhibit photo-inhibition. The rapid light curve fitting of the three macroalgae revealed some differences in the photosynthetic parameters among them. *Hizikia fusiformis* exhibited higher light tolerance than the other two species in light energy use efficiency (January: 0.3; May: 0.29) and semi-saturated light intensity (352.21 μmol (m^−2^s^−1^)) (Table 1).

### 3.3. Diurnal Variation of Effective Quantum Yield of the Macroalgae

**Figure 4:**
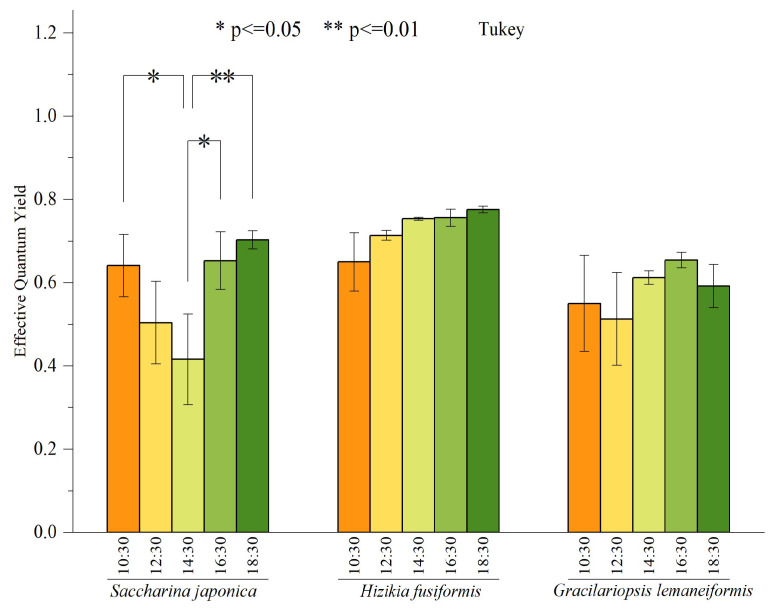
Daily variation of effective quantum yield of three macroalgae. Bars represent average values; error bars denote standard errors (*n =* 3). The asterisks above the bar indicate the significance of difference (one-way ANOVA, Turkey’s test, * *p* < 0.05, ** *p* < 0.01).

In May, we conducted measurements every 2 h to record the daily variation of the effective quantum yield *(F_v_*′/*F_m_*′) for three species of macroalgae. The outcomes of these measurements are depicted in Figure 4. There were no significant differences in *F_v_*′/*F_m_*′ between *Hizikia fusiformis* and *Gracilariopsis lemaneiformis* at different time nodes. However, there were significant differences in *F_v_*′/*F_m_*′ of *Saccharina japonica* at other time nodes. Notably, the maximum *F_v_*′/*F_m_*′ of *Saccharina japonica* was recorded at 18:30, while the minimum value was recorded at 14:30. In general, there is a pattern of initial decline followed by a subsequent recovery.

### 3.4. Rotation Culture Models of the Macroalgae

**Figure 5:**
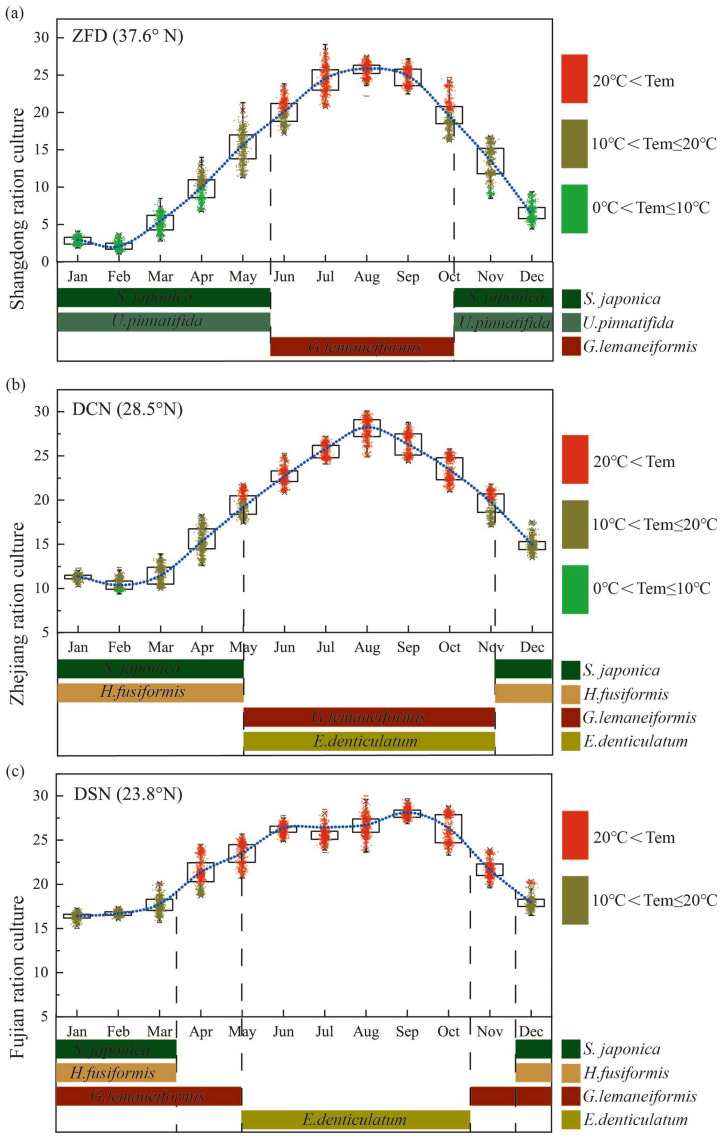
Rotation culture models of macroalgae. Annual water temperature variation at different latitudes in China (observation data in Chinese oceanic stations). (**a**) Shandong ration culture, ZFD: Zhi Fu Dao; (**b**) Zhejiang ration culture, DCN: Da Chen; (**c**) Fujian ration culture, DSN: Dong Shan.

The rotation model for macroalgae was developed based on the variation pattern of water temperature across different latitudes, considering the photosynthetic attributes of the primary cultivated algae, as depicted in Figure 5. For instance, Shandong, representing northern China’s seaweed rotation cultivation (Figure 5a), is characterized by a considerable water temperature range and an extended duration of low temperature. Here, the principal cultivated algal species are *Saccharina japonica* [37,38], *Undaria pinnatifida* [39], and *Gracilariopsis lemaneiformis*. In contrast, Zhejiang province’s macroalgae cultivation mode (Figure 5b) is marked by a brief duration of low temperature. The algae species in rotation predominantly comprises *Saccharina japonica*, *Hizikia fusiformis* [40], *Gracilariopsis lemaneiformis,* and *Eucheuma denticulatum* [41]. In Fujian, as displayed in Figure 5c, the rotation pattern of macroalgae is indicative of a long duration of high temperature, with the rotation algal species mainly consisting of *Saccharina japonica*, *Hizikia fusiformis*, *Gracilariopsis lemaneiformis,* and *Eucheuma denticulatum*.

## 4. Discussion

### 4.1. Response of Photosynthetic Physiological Characteristics of the Three Macroalgae Species to Temperature

Seaweeds are heat-sensitive organisms. Environmental temperature profoundly impacts their enzymatic processes and metabolic functions, such as photosynthesis and respiration [30]. Notably, cold-water and warm-water species exhibit different responses to temperature variations. Cold-water species often experience a decreases in photosynthesis rate and a significant increase in respiration rate when ambient temperature exceeds the optimal range, resulting in the net photosynthesis rate of cold-water algae being less than zero under high-temperature conditions [31].

In this study, a large area of withering occurred at the apical zone of the *Saccharina japonica* in late May. Withering became more serious with the increase in temperature. In late June, the *Saccharina japonica* only retained a part of the holdfast, while the rest all withered. Other relevant studies also report that the loss of individual organisms in *Saccharina japonica* culture during the late stage of temperature stress can be between 45% and 88% [42]. Most *Hizikia fusiformis* withered and decomposed in the first ten days of June and were all withered by middle and late July. In contrast, *Gracilariopsis lemaneiformis* showed promising activity in January (12.3 °C), May (20.3 °C), and September (27.5 °C), and did not wither or decompose.

The relative electron transport rates of cold-water algae *Saccharina japonica* varied significantly differently under the ambient temperature conditions in January and May. These results align with previous experimental results which indicated that the relative electron transport rate of *Saccharina japonica* increases with an increase in temperature within the relatively suitable temperature range. However, when the ambient temperature exceeds the appropriate temperature of *Saccharina japonica*, it leads to death. Pang et al. also demonstrated that when the ambient temperature exceeds 25 °C, the photosynthetic fluorescence parameters of *Saccharina japonica* decrease rapidly, leading to death [12].

Comparted to *Saccharina japonica*, *Hizikia fusiformis* exhibited higher photosynthetic characteristics under different temperatures in January and May, especially in May (20.3 °C). Zou et al. reported that *Hizikia fusiformis* enters the sexual maturation stage when the ambient temperature reaches 19–21 °C, with gametophyte release occurring at temperatures of 27.5–30 °C [31]. The energy distribution of *Hizikia fusiformis* at this stage changes from growth to reproduction. Its higher relative electron transport rate indicates that its photosynthesis provides a higher energy supply. The withering and decomposition of *Hizikia fusiformis* during summer may thus be related to its life cycle. *Gracilariopsis lemaneiformis* exhibited a higher relative electron transport rate in January, May, and September, with the highest relative electron transport rate recorded in September (27.5 °C). Remarkably, it maintained high physiological activity during summer. Yang et al. reported that *Gracilariopsis lemaneiformis* has high nitrogen and phosphorus absorption efficiency and is an excellent target algae species for ecological restoration [43]. The results of field trial culture and simultaneous monitoring of the physiological characteristics of *Gracilariopsis lemaneiformis* under different environmental conditions suggest that it is a suitable species to fill the gap of algae culture in high-temperature waters during summer in northern China.

### 4.2. The Photosynthetic Response of the Three Species of Macroalgae to Light

Macroalgae convert light energy into substances and energy needed for growth, development, and reproduction through the light and dark reaction processes of photosynthesis. Light is the basis of photosynthesis in macroalgae. It affects the light and dark reaction and enzyme activity of macroalgae in the process of photosynthesis [44]. Lack of light limits carbon assimilation and eventually affects the formation of photosynthates in seaweed. In contrast, excessive light causes photo-inhibition to seaweed photosynthesis and even light damage to cells [45]. Different macroalgae have different sensitivity to light because of their morphological differentiation and tissue structure [46].

In this study, the diurnal variation of the effective quantum yield of *Saccharina japonica* decreased at first and then increased. This finding suggested that *Saccharina japonica* has a self-regulation mechanism when the ambient light is less than the saturated light intensity (*E_m_*). Moreover, the photosynthetic reaction center of *Saccharina japonica* does not cause irreversible damage, and its photosynthetic reaction center can recover after adapting to environmental conditions. Previous studies postulate that the photo-regulation mechanism of *Saccharina japonica* is associated with the open state of electron gates in photoreaction center II (PSII) during photosynthesis [47]. Part of the electronic gates of PSII is closed under the continuous irradiation of strong light. PSII transports much of the light energy into heat dissipation to protect the photoreaction, thereby decreasing the actual photochemical efficiency. Notably, the PSII reaction center does not release electrons when the seaweed is in the dark state, and all the electron gates are open, causing the corresponding photochemical efficiency to reach the maximum. In this study, *Hizikia fusiformis* and *Gracilariopsis lemaneiformis* did not show a diurnal changing trend of effective quantum yield. This finding is consistent with the research results of other researchers that report that both algae species have a high tolerance to strong light and that they can adapt to the surface bright-light culture conditions in the actual culture process [23,27,48,49].

Continuous exposure to strong light when the ambient light intensity exceeds the saturated light intensity of macroalgae can cause irreversible photo-damage to the photosystem and result in photo-inhibition (*β* > 0). In this study, *Saccharina japonica* exhibited photo-inhibition in the light curves in January and May, with the photo-inhibition phenomenon being most serious during the seedling stage in January (*β* = 0.05). Hwang et al. reported that the higher light sensitivity and photo-inhibition phenomenon of *Saccharina japonica* is closely related to the tissue structure of its leaves [50]. In the large-scale seaweed culture process, the transparency and depth of the culture water affect the light intensity received on the surface of the algae. The saturated light intensity and light suppression parameters of *Saccharina japonica* thus provide a reference for farmers to arrange the depth of seedling culture to ensure the survival rate of the seedlings.

Optional arrangement of seaweed culture density can maximize algae production per unit area. Light is one of the main factors restricting the culture density of macroalgae. The differences in light energy utilization efficiency among different algae species provide a reference basis for seaweed culture density. In this study, variations in light energy utilization efficiency among the three macroalgae in different seasons were observed, influenced by complex environmental factors. This suggests that the culture density should be planned based on macroalgae growth and adaptation to environmental conditions to maximize the yield per unit culture area.

### 4.3. Design and Application of Rotation Culture Model in Large Seaweeds

**Figure 6:**
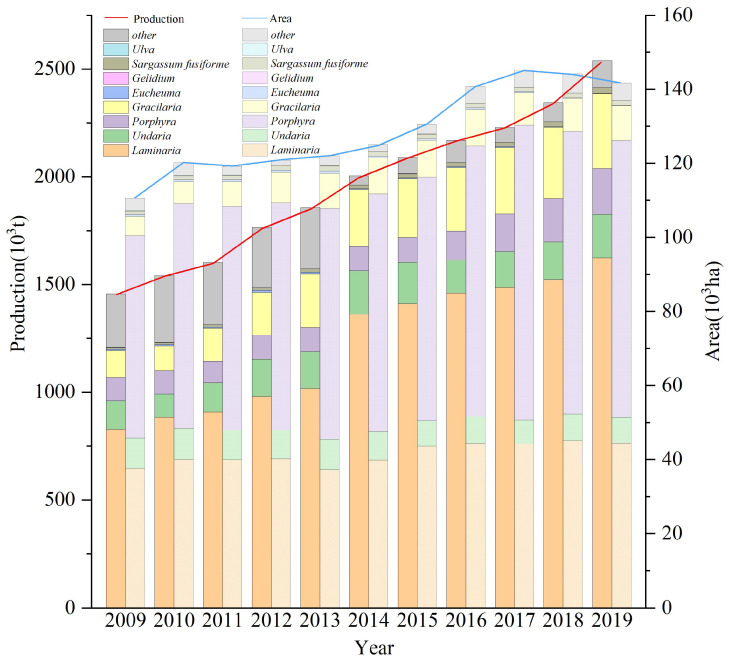
Temporal changes of different aquaculture seaweed productions and farming areas in China between 2009 and 2019 (data from *China Fishery Statical Yearbook*, 2009–2019).

Seaweed culture in China ranges from N 39° to N 22°, with *Saccharina japonica* culture accounting for about 31% of the total algae culture area (Figure 6, *China Fishery Statistical Yearbook* 2009–2019). The period of *Saccharina japonica* culture is usually when the water temperature is below 20 °C. Consequently, China’s algae culture industry thus generally has different degrees of empty window periods from north to south. There are also significant differences between the north and south aquaculture sea areas in the water temperature cycle. It is thus essential to establish a comprehensive algae rotation model based on the water temperature conditions in the south and north. This paper integrates the physiological characteristics of macroalgae with different ecological attributes. Figure 5 outlines the established aquaculture model using Shandong, Zhejiang, and Fujian as examples, based on the environmental and seasonal characteristics of the water temperature of each aquaculture area and the photosynthetic physiological characteristics of each alga. This study focused on Xiapu and other areas in Fujian because of the significant temperature difference between the north and the south of Fujian. There were significant differences in the breeding mode in Xiapu because of the generally high annual water temperature in Dongshan and other southern algae breeding areas in Fujian.

The large seaweed rotation model based on the photosynthetic and ecological characteristics of seaweed is a sustainable development model that uses ecological principles and comprehensively considers the economy, environment, and niche space, thus providing continuous productivity across seasons. The model integrates regulation of the water environment and food web feed supply throughout the year, such as in integrated multi-trophic aquaculture (IMTA) and the construction of seaweed farms. The improvement of water quality also provides bait for fishery resources in aquaculture sea areas. Studies postulate that diversified farming can interrupt the virus transmission cycle and help producers reduce and manage disease risks [51]. The multi-algal species rotation mode and disease control are thus essential for the algae farming industry.

Marine ranches are ecological projects of biological resource conservation and ecological environment restoration. They play a significant role in habitat creation and resource conservation. Seaweed farms are an important part of the marine ranch ecological restoration project. They play a supporting role in the supply of primary productivity, water environment regulation, and habitat creation, and are an indispensable part of the ecological function of marine ranches [52]. Relevant studies also postulate that seaweed farms have made outstanding contributions to the conservation of fishery resources, especially for fish larvae, which directly affect the replenishment of fish populations [5]. However, most sea areas, such as the East China Sea, are characterized by low water transparency and lack of suitable seaweed attachment bases, thus making it challenging to restore natural seaweed farms. The large-scale seaweed raft culture structure provides ideas for solving the challenges of seaweed farm restoration in the East China Sea. The findings of this study, combined with our previous extensive investigation and research work, suggest that the time complementarity should be considered when constructing artificial seaweed farms under the ecological restoration framework of marine ranch. The stability of the project is mainly based on the conservation of biological resources, the restoration of the ecological environment, and the economic benefits for fishermen. The large-scale seaweed rotation mode provides good theoretical support for constructing artificial seaweed farms. It provides a solution to restoring seaweed farms in the East China Sea and a tool for ecological restoration.

## 5. Conclusions

This study carried out a trial culture study in aquaculture sea areas and monitored the photosynthetic characteristics of *Saccharina japonica*, *Hizikia fusiformis*, and *Gracilariopsis lemaneiformis* at different times. The study proposes a rotation model of macroalgae, covering the whole year based on the current ecological habits of large cultured algae, combined with the annual water temperature changes in different latitudes in China, taking Shandong, Zhejiang, and Fujian as examples. It provides a solution to the limitations of the production and ecological effects of single algal culture. This study only considers the suitability of macroalgae photosynthetic and physiological characteristics to field temperature and light conditions. Future studies should thus focus on the suitability of macroalgae photosynthetic and physiological activity on environmental factors, such as nutrients, flow field, light quality, and trace elements, to have a holistic conclusion, especially on the rotation model.

## Figures and Tables

**Table 1:** Rapid light curve fitting parameters of three macroalgae.

Species	Parameters	January	May	September
*Saccharina japonica*	*α*	0.23 ± 0.04 ^a^	0.22 ± 0.04 ^a^	-
	*β*	0.05 ± 0.02 ^a^	0.01 ± 0.01 ^b^	-
	*rETR_max_*	43.58 ± 9.59 ^b^	58.2 ± 8.78 ^a^	-
	*E_k_*	187.33 ± 66.05 ^b^	264.91 ± 84.17 ^a^	-
	*E_m_*	328.66 ± 39.2 ^b^	970.9 ± 148.8 ^a^	-
	*R^2^*	0.86	0.79	-
*Hizikia fusiformis*	*α*	0.29 ± 0.03 ^a^	0.3 ± 0.03 ^a^	-
	*β*	0	0	-
	*rETR_max_*	70.45 ± 4.48 ^b^	106.75 ± 8.62 ^a^	-
	*E_k_*	238.85 ± 35.08 ^a^	352.21 ± 54.13 ^a^	-
	*E_m_*	1201.72 ± 211.15 ^a^	-	-
	*R^2^*	0.91	0.96	-
*Gracilariopsis lemaneiformis*	*α*	0.29 ± 0.02 ^a^	0.33 ± 0.05 ^a^	0.19 ± 0.02 ^b^
	*β*	0	0	0
	*rETR_max_*	75.33 ± 4.37 ^b^	74.33 ± 6.72 ^b^	93.07 ± 13.21 ^a^
	*E_k_*	263.46 ± 32.95 ^b^	224.8 ± 47.6 ^b^	495.08 ± 111.55 ^a^
	*E_m_*	1095.08 ± 84.7 ^a^	973.29 ± 130.63 ^a^	-
	*R^2^*	0.94	0.89	0.96

Notes: different letters mean significant difference (*p* < 0.05).

## Data Availability

The original contributions presented in the study are included in the article, and further inquiries can be directed to the corresponding author.
